# Supplementary material for: Stimulated emission depletion microscopy with a single depletion laser using five fluorochromes and fluorescence lifetime phasor separation
Source: Sci Rep. 2022 Aug 18;12:14027. doi: 10.1038/s41598-022-17825-5 (PMC9388687; doi:10.1038/s41598-022-17825-5)
Supplement: Supplementary file 1 — Supplementary Figures. [file 41598_2022_17825_MOESM1_ESM.docx]

Supplemental Figure 1


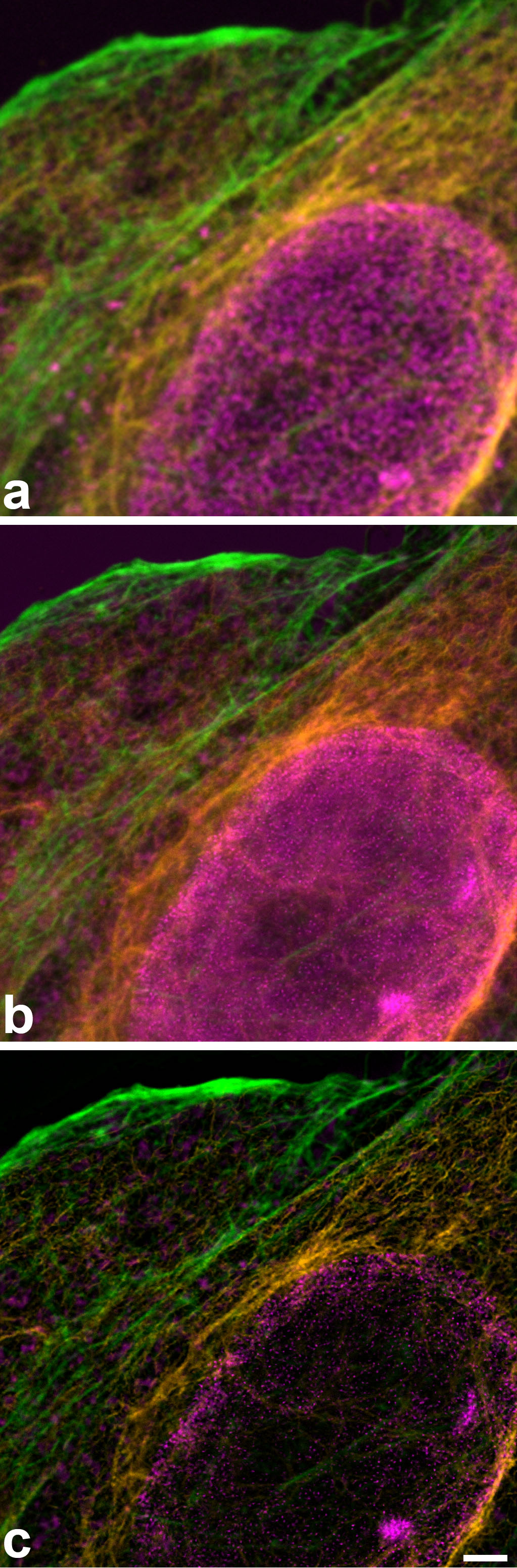


Supplemental Figure 1: Full images of the composition shown in Figure 3: Three color images with spectrally separated SPY555-actin (green) and antibody stainings against vimentin (Alexa Fluor 594, orange) and nuclear pores (CF680R, magenta), all depleted with 775 nm. (a) confocal, (b) STED (c) Tau-STED. Scale bar: 2 µm.

Supplemental Figure 2:


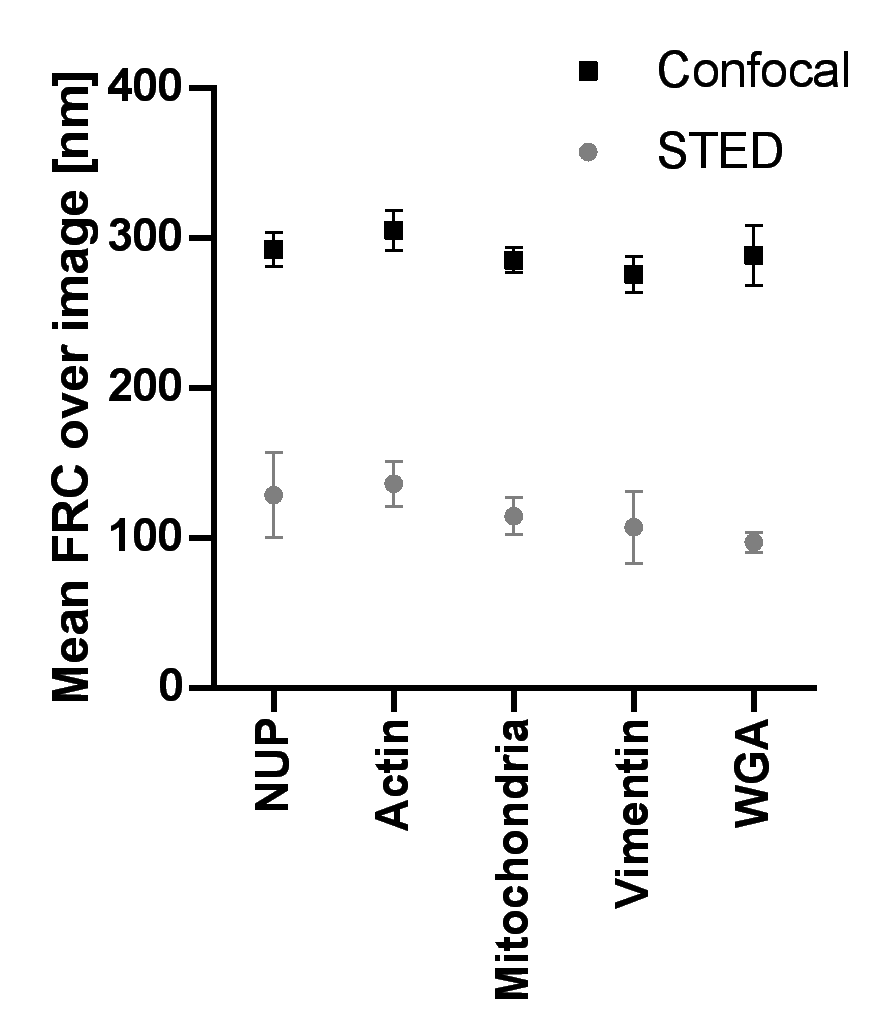


Supplemental Figure 2: Resolution determined by Fourier Ring Correlation in phasor separated 5 color images. Matching confocal and STED images were analyzed (each group n=5). Mean values and standard deviations are indicated.

Supplemental Figure 3:


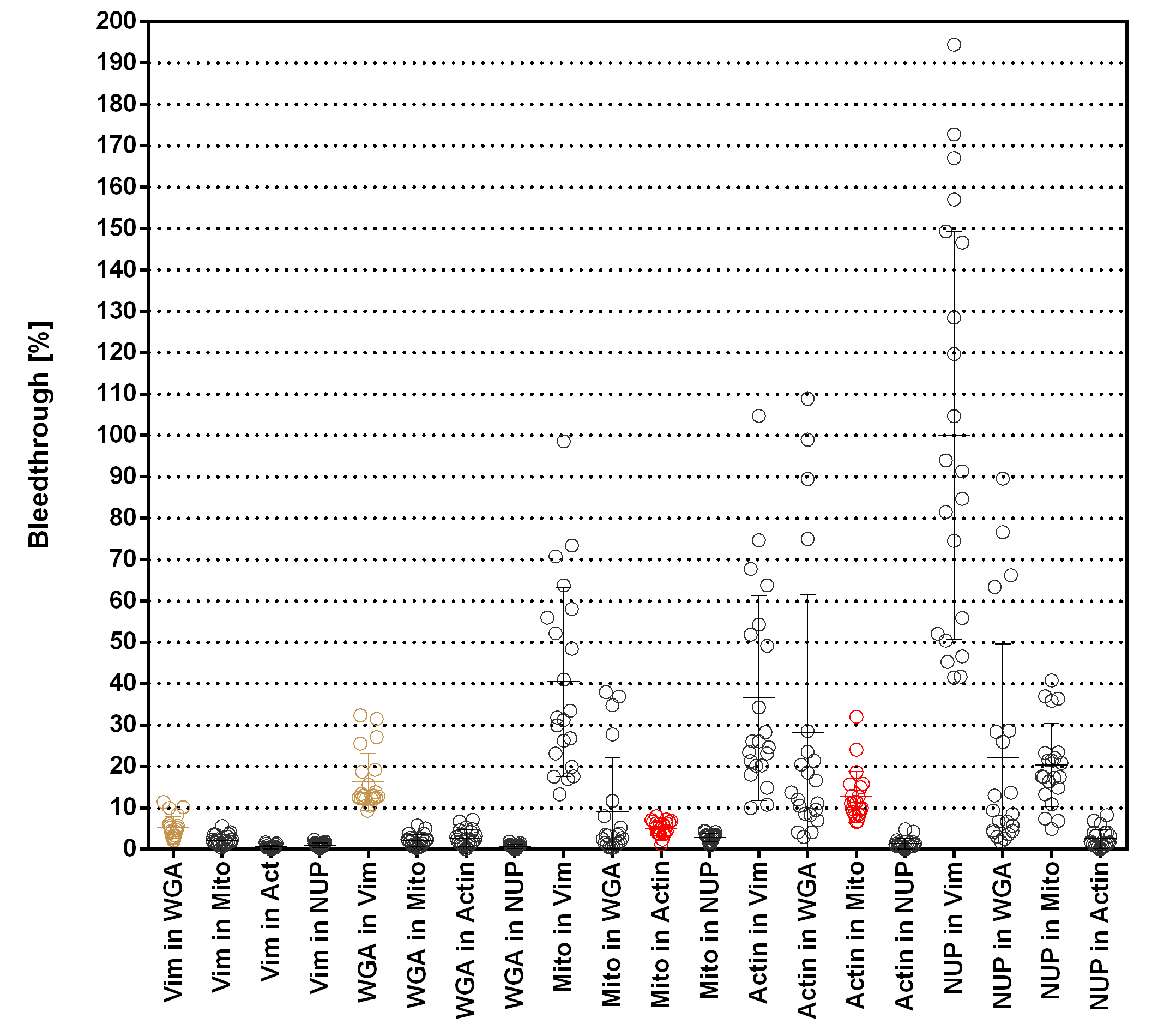


Supplemental Figure 3: Bleed-through values for spectrally and lifetime separated signals in five color FLIM-STED images, n=21 image sets. Bleed-through between images generated by phasor separation from the same raw images are shown in the same color, all others in black. Bleed-through values are dependent on general signal intensities in the various images and thus can reach very high values, here for NuP in vimentin. This problem is further explained and visualized in Supplemental Figure 4.

Supplemental Figure 4:


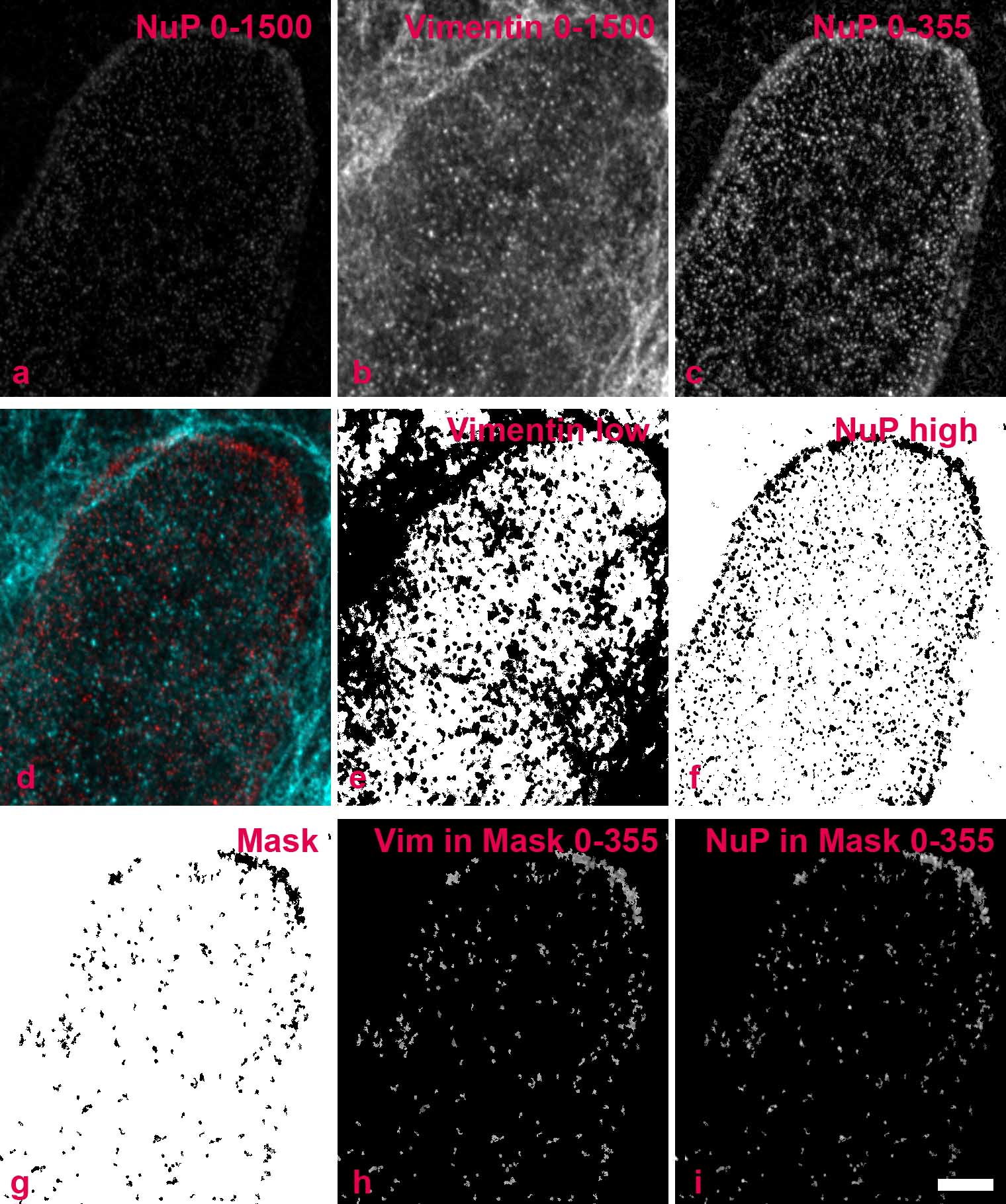


Supplemental Figure 4: Visualization of image processing operations for bleed-through and crosstalk calculations from nuclear pore (NuP) signal into the vimentin channel. This particular example also demonstrates why bleed-though is not a good measure, and that normalized contribution values are more meaningful. Vimentin signals were much brighter than nuclear pore signals, causing a high “bleed-through”-value for NuPs in vimentin (Suppl. Fig. 3). Only a part of STED-images is shown.

a) NuP image scaled to display gray levels between 0 and 1500 (0= black, 1500=white). The signal appears weak. b) Vimentin image with the same scaling used in (a), the signal is well recognizable. c) Image as in (a) but now scaled to display 0-355 gray levels. d) Overlay of (b) and (c). The punctuate pattern of vimentin on the nucleus is different from the NuP pattern. e) Mask generated by applying a low threshold value to the vimentin image to exclude all areas above the threshold (those areas with vimentin signal = black areas). f) Mask generated by applying a high threshold value to the NuP image to define all areas with intense NuP signal (= black areas). g) Final mask generated by subtracting (e) from (f). This mask was used to generate a region of interest (ROI) in the vimentin and NuP-channels for further calculations. h) Signal of the vimentin channel in the ROI only, scaled to display gray levels between 0-355 (compare (b) and (c)). Since the vimentin signal was excluded from the ROI, this remaining signal is the supposed spillover of the NuP signal into the vimentin channel. i) Signal of the NuP channel in the ROI only, scaled to display gray levels between 0-355. Scale bar: 2 µm for all images.

Vimentin signals have a much higher gray level than NuP signals. The vimentin channel background too is rather high compared to the NuP signal. This leads to an elevated estimate for the spillover of NuP into the vimentin channel: The bleed-through of NuP signal into the vimentin channel was calculated as the ratio vimentin/NuP where “vimentin” is the supposed bleed-through of the NuP signal (see above). For this particular image set, average intensity values of the ROI of the whole images (not just the clippings shown here) were 169 for vimentin and 113 for NuP, resulting in a bleed-through value of 149%, i.e. the calculated bleed-through had a higher gray level than the signal from which it originated.

To avoid such obvious misdirection caused by different intensity levels in different images, contribution values were normalized to the 99-percentile gray levels of both images. In the given example, for the complete images the 99-percentile values were 862 for vimentin and 105 for NuP. Contribution was calculated as 149%*(105/862)=18%.
